# Lung Involvement in Patients with Ulcerative Colitis: Relationship between Exhaled Nitric Oxide and Lung Function

**DOI:** 10.3390/jcm13020354

**Published:** 2024-01-08

**Authors:** Beatrice Ragnoli, Tiziana Cena, Patrizia Pochetti, Patrizia Pignatti, Mario Malerba

**Affiliations:** 1 Respiratory Unit, S. Andrea Hospital, 13100 Vercelli, Italy; beatrice.ragnoli@aslvc.piemonte.it (B.R.); patrizia.pochetti@aslvc.piemonte.it (P.P.); 2 Epidemiological Observatory Service, ASL VC, 13100 Vercelli, Italy; tiziana.cena@aslvc.piemonte.it; 3 Allergy and Immunology Unit, Istituti Clinici Scientifici Maugeri IRCCS Pavia, 27100 Pavia, Italy; patrizia.pignatti@icsmaugeri.it; 4 Department of Traslational Medicine, University of Eastern Piedmont, 28100 Novara, Italy

**Keywords:** ulcerative colitis, extended NO analysis, lung function tests, diffusing capacity, clinical disease activity

## Abstract

Ulcerative colitis (UC) is characterized by immune system dysregulation with frequent extraintestinal manifestations, including airway involvement. A reduction in CO diffusing capacity and functional alterations in small airways have been described. An extended analysis of fractional exhaled nitric oxide (FeNO) may distinguish the sites of production, and the presence of small airway inflammation may be a useful, non-invasive marker for patient follow-up. The aim of our study was to compare the PFTs as well as FeNO and CANO values of UC patients with different clinical disease activities and healthy subjects to reveal lung function abnormalities and the presence of subclinical airway inflammation. We enrolled 42 adult outpatients at different clinical activity stages of UC (39 ± 13 years) and a healthy control group of 41 subjects (29 ± 3 years). C-reactive protein (CRP) and FeNO values at different flows (50,100, and 200 mL/s) were collected. All patients performed pulmonary function tests (PFTs) with static volumes and diffusing capacity (DLCO). FeNO and CANO values were significantly increased in UC patients when compared with controls (*p* = 0.0008 and *p* < 0.0001, respectively) and were proportional to disease activity (FeNO class 3: 28.1 ppb vs. classes 1–2: 7.7 ppb; CANO values class 3: 8.6 ppb vs. classes 1–2: 2.7 ppb (*p* < 0.0001)). TLC and DLCO were significantly reduced in severe (Mayo 3) UC patients (*p* = 0.010 and *p* = 0.003, respectively). The results of this study show significant lung functional abnormalities in UC patients and suggest the presence of airway inflammation directly correlated with disease activity, suggesting the need for an integrated approach in routine assessment.

## 1. Introduction

Ulcerative colitis (UC) is a chronic inflammatory bowel disease (IBD) characterized by immune system dysregulation, leading to significant local tissue inflammation and frequent extra-intestinal manifestations. These include active airway involvement [1,2], which may worsen disease prognosis [3]. Clinical and subclinical pulmonary abnormalities, such as functional alterations, especially in small airways, and impaired diffusing capacity for carbon monoxide, have been documented in patients with UC and other IBDs. These abnormalities may indicate the presence of a subclinical inflammatory state, even in the absence of symptoms. However, evidence regarding the association of pulmonary function tests (PFTs) with disease activity remains controversial [4]. Some reports have indicated that these abnormalities also persist and have been observed during clinical remission of the disease, suggesting a latent subclinical inflammatory reaction [5]. Among recent, straightforward, non-invasive methods for assessing airway inflammation, fractional exhaled nitric oxide (FeNO) measurement has proven effective in detecting and safely monitoring airway inflammatory status in various clinical conditions, in both adults and children [6]. Standardized procedures for measuring FeNO concentration have gained approval from the American Thoracic Society (ATS) and the European Respiratory Society (ERS) [7,8]. Moreover, advancements in the field of NO dynamics, exemplified by extended NO analysis, have provided valuable insights into the characterization and measurement of airway inflammation [9]. In particular, extended NO analysis, which involves measuring FeNO levels at multiple expiratory flow rates, enables the estimation of alveolar NO concentration (CANO) based on the total amount of FeNO [10]. This methodology facilitates the identification of the specific sites responsible for FeNO production and offers potential insights into the presence of small airway inflammation. Thus, FeNO assessment can directly reflect airway inflammation and holds promise as a non-invasive marker for monitoring these patients during their follow-up. In line with the aforementioned advancements, the primary objective of the current study was to conduct a comparative analysis of PFTs and FeNO and CANO values between a cohort of UC outpatients displaying varying clinical disease activities and a control group of healthy subjects. This analysis aimed to uncover potential lung function abnormalities and evaluate the presence of subclinical airway inflammation in UC patients. Furthermore, we sought to determine whether a correlation existed between inflammatory markers, the primary sites of NO production, and clinical disease activity. Lastly, we conducted a correlation analysis between FeNO, CANO, and systemic inflammation indices, such as C-reactive protein (CRP).

## 2. Materials and Methods

### 2.1. Population

For this study, we enrolled a cohort of 42 adult outpatients (mean age, 39.0 ± 13.3 years), with different clinical activity stages of UC. These subjects had no concurrent pulmonary diseases and attended the outpatient gastrointestinal clinic at the S. Andrea Hospital in Vercelli, Italy. In addition, we included a control group of 41 healthy subjects (mean age, 29.5 ± 3.8 years), who were non-smokers and without a history of atopy. These subjects were selected from the general population and matched for age and sex. The diagnosis of UC was based on a combination of past medical history, physical examination, and radiological, endoscopic, and histological findings. We also recorded demographic information, clinical history, disease duration, prescribed medications, and disease extent assessment. The enrolled patients did not report any symptoms or signs of lung disease, such as cough, sputum, dyspnea, and hemoptysis. We implemented exclusion criteria to account for potential factors that might influence FeNO levels, including cigarette smoking, atopy assessed via skin-prick tests to common inhalant allergens, respiratory tract infections over the previous 6 weeks, and lung diseases, such as asthma, chronic obstructive pulmonary disease (COPD), cystic fibrosis, bronchiectasis, and respiratory failure. Upon enrolment in this study, each patient belonging to the UC group received tailored treatment regimens involving topical and/or systemic administration of anti-inflammatory drugs (i.e., mesalazine), biological drugs (i.e., infliximab and adalimumab), and immunosuppressants (i.e., methotrexate and azathioprine). These treatment protocols were individualized, continuously monitored, and adjusted as needed. This study was approved by the Institutional Review Board CE 67/20, in accordance with the principles of the Declaration of Helsinki. All patients and control subjects provided informed written consent before participating in the study.

### 2.2. Study Design

We performed a single-center, controlled, observational study. At the time of enrolment, all UC patients underwent a thorough examination and completed a specifically designed questionnaire to detect the presence and extent of both intestinal (i.e., abdominal pain, fever, frequency of defecation, and presence of blood in the feces) and pulmonary (i.e., cough, sputum, dyspnea, and hemoptysis) symptoms. In addition, we gathered demographic data and clinical history and conducted macroscopic and histological evaluation of the disease. We also documented the duration and severity of the latter, medications used, and any existing co-morbidities. On the day of the initial FeNO measurement, a blood sample was collected for CRP analysis. Forty-one control subjects were subsequently enrolled from healthy volunteers whose medical history and physical exam were negative for UC. Following enrolment, all patients and control subjects underwent assessment, which included FeNO evaluation, lung function testing, comprising maximum expiratory flow–volume curves and body plethysmography, as well as measurements of diffusing capacity for carbon monoxide (DLCO).

### 2.3. Disease Activity

The activity of UC was assessed by means of the colitis activity index (CAI), which classifies patients into three classes based on increasing clinical severity (1, 2, and 3) based on a score ranging from 0 to 12. UC patients who scored below 2 were considered to be in clinical remission, while those with scores exceeding 6 were identified as having active disease. More precisely, the scoring criteria were as follows: <2 for remission, 3–5 for mild activity, and 6–12 for moderate to severe activity [11]. In this study, we considered class 1 + 2 as mild–moderate disease and class 3 as severe disease. Clinical evaluations were conducted, and therapy was tailored to individual patient needs to ensure optimal disease management.

### 2.4. Pulmonary Function Tests

All patients underwent PFTs, which included forced expiratory volume in one second (FEV1), forced vital capacity (FVC), and static volume measurements, carried out using body plethysmography, as well as DLCO, performed through single-breath measurements. These tests were conducted following the completion of FeNO measurements, in accordance with established ATS/ERS guidelines [12]. Spirometry and maximal full flow–volume curves were obtained using a pneumotachograph with volume integrator (1070 MGC; CAD/Net system; Medical Graphics Corporation, St. Paul, MN, USA). The best values were selected from a minimum of three acceptable measurements. The results were expressed as a percentage of predicted normal values adjusted for sex, age, height, and weight following technical standards for spirometry [13]. DLCO adjustments for lung volumes and hemoglobin values in UC patients were made in accordance with ATS/ERS recommendations [14]. Global Lung Function Initiative (GLI) reference equations for spirometry, diffusing capacity, and lung volumes were used to define the expected range of values in the healthy control group [15,16].

### 2.5. FeNO and CANO Measurements

FeNO levels were assessed before spirometry via the single-breath method, using a high-resolution chemiluminescence NO analyzer (Ecomedics AG CLD 77 AM; Ecomedics; Durnten, Switzerland), with a 0.06 parts per billion (ppb) detection limit and an upper measuring range limit of 100 ppb. Measurements were performed at various flow rates, following ATS/ERS recommendations and employing a standardized procedure for the online measurement of FeNO in adults [7,8,9]. Single FeNO measurements were taken at a flow rate of 50 mL/s, followed by measurements at multiple flow rates of 50, 100, and 200 mL/s. For each flow rate, the mean value of three measurements was used. A mathematical approach based on a two-compartmental model was applied to distinguish NO generated in the distal airways (i.e., CANO) [17]. A CLD 77 analyzer was calibrated at 0 ppb and 100 ppb following the manufacturer’s instructions. Daily calibration was performed using a certified NO mixture (96 ppb) in nitrogen (Messer S.p.A.; Collegno-TO, Italy). Ambient air was monitored for NO concentration prior to data collection, and measurements were only taken when ambient NO levels were below 10 ppb. Establishing a definitive normal range for FeNO is notoriously challenging, and reference values for FeNO have been defined for various groups, including healthy individuals, the elderly, and children [18,19,20,21,22]. For healthy individuals, the upper limit of FeNO50 has been defined by the National Health and Nutrition Examination Survey (NHANES). Their data indicate that FeNO levels in individuals aged 12 to 80 typically fall within the range of 3.5 to 39 ppb, representing the 5th to 95th percentile [23]. In a recent review, the application of multiple regression modeling indicated normal values of FeNO in never-smoking adults, ranging from 24 to 53 ppb. The data showed that in an unselected population, the distribution of FeNO was skewed to the right. Therefore, the authors concluded that reference values derived from a ‘normal’ population may be less useful than specific cut-off points for patients with airway disease or respiratory symptoms [24]. Considering the data previously discussed, we regarded all FeNO readings below 25 ppb at a constant flow rate of 50 mL/s as normal, without clinical relevance. All tests were conducted in the morning, at the same time, with patients required to have an empty stomach. Patients treated with biological drugs (i.e., infliximab and adalimumab) were carefully examined prior to each injection.

### 2.6. CRP Collection

CRP values were determined from venous blood samples of UC patients drawn as part of routine clinical assessment, which comprised a full blood count and CRP measurement. No blood samples were drawn from the healthy control group.

### 2.7. Endpoints and Statistical Analysis

The primary objective of this study was to compare lung function parameters as well as FeNO and CANO levels in UC patients exhibiting different clinical disease activities with those from a healthy control group. In addition, in the UC subgroup, we assessed the correlation between FeNO, CANO, and systemic inflammation indices, such as CRP. Data were presented, with frequencies and percentages for categorical variables, as mean value ± standard deviation and median (1st–3rd quartile) for continuous variables. Chi-squared test was used to evaluate the association between the categorical variables, such as sex, UC/controls, and Mayo score. The mean differences between lung function parameters and inflammatory markers in the UC group and the healthy control group as well as Mayo score groups were assessed with the two-sample *t*-test (Student’s *t*-test). A multivariate linear regression model was used to account for possible confounding variables, such as sex and age. The Pearson correlation coefficient was calculated to examine the correlation between FeNO, CANO, and systemic inflammation indices (CRP) in the UC subgroup. A *p*-value of <0.05 was considered significant. Statistical analyses were performed using SAS 9.4 (SAS Institute, Cary, NC, USA) software.

## 3. Results

### 3.1. Baseline Characteristics of Study Participants

The baseline clinical characteristics of the study population are reported in Table 1. A total of 83 patients were enrolled, consisting of 42 UC patients (50.6%) and 41 healthy subjects (49.4%). Among the entire study cohort, 39/83 subjects were males (46.9%), with 22/42 being UC patients (52.4%) and 17/41 being healthy controls (41.4%). The mean age for UC patients was 39 years, while that of healthy controls was 29.5 years (*p* < 0.0001). The body mass index (BMI—body mass index) did not show any significant difference between the two groups, with a mean value of 22.6 in UC patients and 22.7 in healthy subjects. Regarding disease activity in the UC group, 3 subjects (7.1%) displayed mild disease activity (Mayo score: 1), 13 subjects (31%) had moderate disease activity (Mayo score: 2), and 26 patients (61.9%) presented with severe disease activity (Mayo score: 3). The mean value of CRP in the UC group was 24.1 ± 23.3 mg/dL.

### 3.2. Pulmonary Function Tests

**Figure 1:**
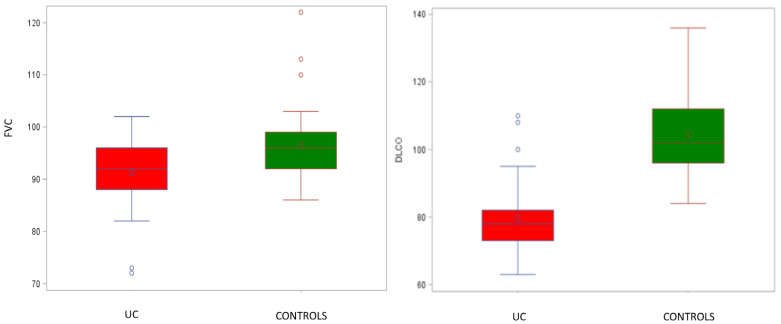
Comparison between forced vital capacity (FVC) and monoxide diffusing capacity (DLCO) in patients with UC and control group. There is a statistically significant reduction in FVC and DLCO in the two groups. Abbreviations: UC—ulcerative colitis; FVC—forced vital capacity; DLCO—diffusing capacity for carbon monoxide; %—percentage of predicted; o—mean values.

None of the patients in either group exhibited an FEV1/FVC ratio below 70% or an FEV1 value lower than 70% of the predicted one, indicating the absence of airway obstruction. UC patients had a mean FEV1/FVC ratio of 82.0 ± 5.9%, a mean predicted FEV1 of 95.8 ± 9.6%, and a mean predicted FVC of 91.3 ± 6.7%. In contrast, the control group showed a mean FEV1/FVC ratio of 84.5 ± 3.1%, a mean predicted FEV1 of 100.2 ± 9.2%, and a mean predicted FVC of 96.5 ± 7.3%. We observed a reduction in FVC values in the UC group to the limits of statistical significance when adjusted for age, compared to healthy subjects (*p* = 0.005; Figure 1). However, there were no statistically significant differences between FEV1 and FEV1/FVC measurements in the two groups (*p* = 0.52 and 0.53, respectively). Regarding lung volumes, no restrictive pattern was observed, with TLC values not falling below 80% of the predictive values in both groups. Nevertheless, it is important to point out that the predictive TLC values were lower in the UC group, with a mean value of 92.0 ± 11.1%, compared to those of the control group, which had mean values of 98.8 ± 12.2%, even though, when adjusted for age, they did not reach statistical significance. The predicted DLCO values were markedly lower in the UC group, with a mean predictive value of 79.7 ± 10.6%, compared to the control group, where it was 104.4 ± 12.1% (*p* < 0.0001). It is also worth mentioning that DLCO values below 80% of the predicted value indicate a mild reduction in CO diffusion, which is considered clinically relevant for these patients (Table 2 and Figure 1).

### 3.3. FeNO Measurements

**Figure 2:**
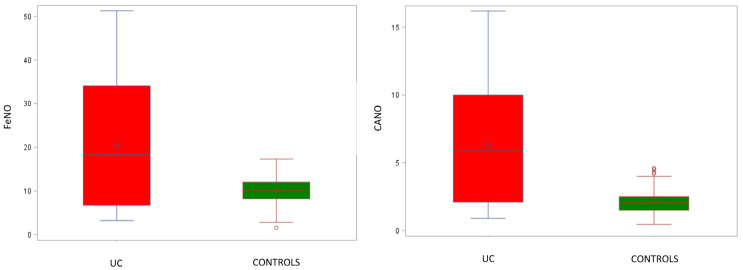
Comparison between bronchial (FeNO) and alveolar (CANO) nitric oxide in patients with UC and control group. FeNO and CANO levels are significantly increased in the UC group. Abbreviations: UC—ulcerative colitis; FeNO—fractional exhaled nitric oxide; CANO—alveolar concentration of nitric oxide; o—mean values.

FeNO values were significantly increased in UC patients in comparison with those from healthy controls. The mean bronchial FeNO at 50 mL/s was 20.4 ± 14.7 ppb for UC patients, whereas healthy controls had a mean of 10.3 ± 3.4 ppb (*p* = 0.0008). A similar trend was observed for CANO levels, which were also significantly higher in UC patients. Specifically, the mean CANO levels were 6.3 ± 4.5 ppb for UC patients, whereas healthy controls displayed a mean of 2.2 ± 1.0 ppb (*p* < 0.0001; Table 2 and Figure 2).

### 3.4. Lung Function, FeNO, CRP, and Correlation with Clinical Disease Activity

**Figure 3:**
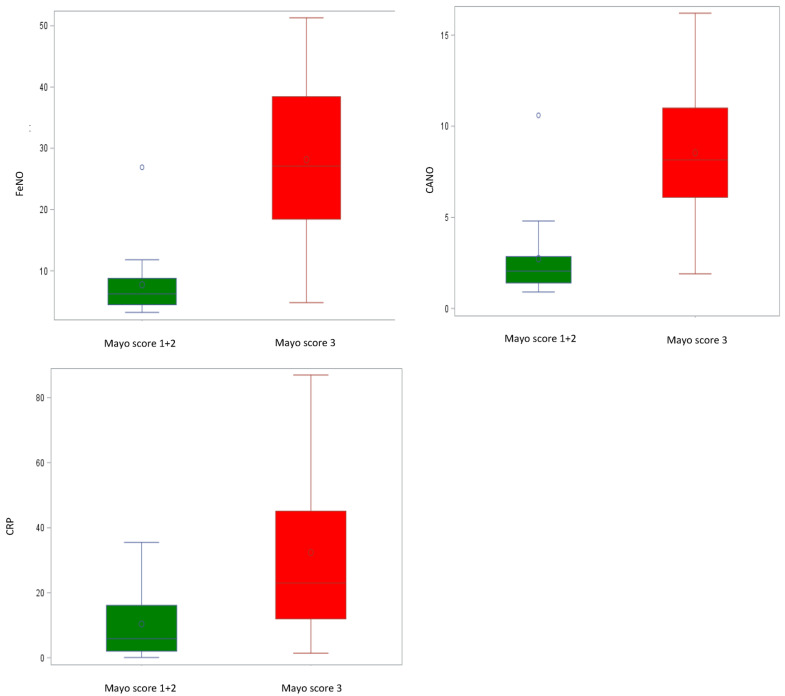
Comparison between disease activity, bronchial (FeNO), alveolar (CANO) nitric oxide levels and CRP levels in patients with UC. FeNO and CANO levels and CRP levels are significantly increased in UC patients with active disease (Mayo score 3). Abbreviations: UC—ulcerative colitis; CRP—C-reactive protein; FeNO—fractional exhaled nitric oxide; CANO—alveolar concentration of nitric oxide; o—mean values.

The correlation between pulmonary function and inflammatory indices with clinical disease activity in the subgroup of UC patients was evaluated. Table 3 presents lung function, CRP, and exhaled NO measurements for the UC subgroup categorized by disease activity (Mayo score). Notably, we observed a significant increase in FeNO, CANO, and CRP values that corresponded with disease activity, especially in its severe form. The mean FeNO values for class 3 (severe) were 28.1 ± 12.9 ppb compared to classes 1–2 (mild) at 7.7 ± 5.7 ppb (*p* < 0.0001). Similarly, for CANO, the mean values for class 3 were 8.6 ± 4.1 ppb, while classes 1–2 had mean values of 2.7 ± 2.3 ppb (*p* < 0.0001). The CRP values followed the same trend, with mean values of 32.5 ± 24.9 mg/dL for class 3 compared to 10.4 ± 11.4 mg/dL for classes 1–2 (*p* = 0.0020). These findings indicate a strong relationship between these markers and disease activity in the UC subgroup. It is worth noting that in severe cases of UC (class 3), the FeNO measurement exceeded the upper normal limit, reaching pathological values, even in the absence of respiratory symptoms or conditions (Figure 3). Finally, we observed a statistically significant reduction in TLC after adjusting for age and a significant reduction in DLCO values in severe UC patients, indicating a correlation between clinical disease activity and the functional parameters TLC and DLCO (Table 3).

## 4. Discussion

The results from our study reveal the remarkable subclinical inflammatory involvement of the lungs in UC patients related to disease activity, as evidenced by significantly elevated FeNO and CANO levels compared to those observed in healthy controls. Furthermore, our findings show significant alterations in lung volumes and DLCO in these patients, which is consistent with the hypothesis that mechanisms affecting both central and distal airways may be at play. To the best of our knowledge, our study is the first to comprehensively assess bronchial and distal airway inflammation through extended NO analysis alongside PFTs (e.g., DLCO and lung volumes). IBDs, such as UC, exhibit a wide spectrum of lung involvement, ranging from subclinical abnormalities to airway or interstitial lung disease (ILD) [25]. These lung abnormalities can manifest at various stages of the disease, including at its onset, during active disease [26], or even following surgical interventions. The underlying pathogenesis may be linked to a shared embryonic origin of colonic and respiratory epithelial cells, along with similarities in mucosal immunity, leading to analogous pathogenetic alterations [27]. Previous studies have shown a prevalence of PTF abnormalities ranging from 17% to 55% in UC patients, underscoring the potential to identify occult pulmonary conditions at an early stage using functional indices [28]. In our study, we report a significant reduction in FVC values in the UC group compared to healthy subjects. However, we found no differences between FEV1 and FEV1/FVC measurements in both groups nor did we identify obstructive dysfunction or a correlation with disease activity. In line with our results, previous research has shown isolated reductions in absolute values of FEV1, FVC, and forced mid-expiratory flow (FEF 25–75%) [29,30,31,32,33]. More specifically, obstructive dysfunction was observed at times associated with disease activity [34,35]. Although our study demonstrates a reduction in dynamic lung volumes, particularly FVC, which is indicative of subclinical restrictive ventilatory dysfunction, among UC patients compared to controls, we could not confirm a similar significant reduction in TLC in UC patients, especially when adjusting for age. Nonetheless, FVC may have a clinically relevant impact, being a dynamic lung volume index not influenced by residual volume, hence the importance of assessing it. However, when considering the correlation with disease severity, we observed a notable statistically significant reduction in TLC values among patients with a Mayo score of 3 when compared to patients with scores of 1–2 (*p* = 0.0100). The existing literature presents conflicting findings regarding lung volumes in patients with UC. While most studies have reported increased lung volumes (i.e., TLC, RV, and FRC) in individuals with IBD, which is often linked to disease activity [26,36,37], other studies have shown restrictive patterns and decreased lung volumes in children [32,38]. Several mechanisms might be responsible for the observed alterations in spirometry measurements among these patients. In addition, different mechanisms may overlap to determine the final effect. One hypothesis suggests that a loss of body proteins and a reduction in BMI may indicate poor nutritional status, potentially contributing to reduced spirometry parameters [31,39]. Another explanation relates to an elevated percentage of alveolar lymphocytes, sensitized from the gastrointestinal tract, which may lead to lung alveolitis, thereby altering PFT results [31,40,41,42,43]. Of particular significance in our study is the observed reduction in DLCO. Indeed, our data show a statistically significant decrease in DLCO in UC patients compared to healthy subjects, reaching clinical relevance (78% of predicted values). Furthermore, we could establish a statistically significant correlation between DLCO values and the severity of the disease (*p* = 0.0030), with a discernible reduction in DLCO among patients with a more severe form of the disease. These results are in line with previously reported data in the literature. DLCO stands out as the most frequently reported abnormality in PFTs, in both adults and children with IBD [5,34,44,45,46]. Moreover, several studies have recorded alterations in DLCO consistent with ILD, and these changes have been associated with disease activity [26,32,34,35]. The precise pathophysiological cause of this DLCO reduction remains poorly understood. The integrity of both the lung and the gut epithelium is guaranteed via a sophisticated system of regulatory junctions, which provides a protective barrier to noxious external agents [47]. Current theories propose an imbalance in IBD patients involving an altered immune response to the gut microbiota [48,49,50], disrupting the intestinal epithelial barrier and allowing immunoreactive gut cells to migrate to lung tissues. This migration is then thought to trigger an intense cross-talk between intestinal and pulmonary immune cells [51]. Moreover, inflamed gut mucosa in IBD is associated with increased levels of nonspecific inflammatory mediators, including cytokines, chemokines, growth factors, reactive oxygen radicals, and NO [52]. Furthermore, damage to the gut epithelium determines a decrease in the short-chain fatty acids, produced during bacterial fermentation of dietary fiber, which are important stabilizers of the integrity of both gut and lung epithelia [53]. These factors collectively contribute to inflammation and oxidative stress, which may, in turn, lead to impaired DLCO. The primary finding of our study underscores a substantial increase in FeNO and CANO levels recorded among UC patients when compared to the healthy control group. In addition, we report a statistically significant correlation between the levels of FeNO and CANO and the severity of disease, with higher values observed in UC patients with a Mayo score of 3, indicating severe disease activity. NO plays an important role in many physiological and pathological processes involving the gastrointestinal tract, and it is recognized as a key inflammatory mediator in IBD. Previous research has shown increased NO levels in colonic gases of UC patients in comparison with control subjects [54]. FeNO originates from the airway epithelial cells through the upregulation of inducible nitric oxide synthase (i-NOS) activity. Of note, FeNO has not only been found elevated in patients with IBD but is also positively associated with disease activity [55,56,57,58,59]. However, CANO represents a marker of small airway inflammation, extensively investigated in conditions such as asthma [38,60,61,62,63,64] and other pulmonary diseases [65,66,67,68,69,70,71,72]. Moreover, increased CANO values have been found even in the absence of disease activity, indicating the subclinical involvement of small airways [5]. In our study, we observed that FeNO and CANO values increase in parallel with disease activity, suggesting a correlation between airway inflammation and the clinical severity of intestinal disease. This finding hints at the involvement of alveolar sites (alveolitis) and small vessel compartments of the lung in chronic IBD.

## 5. Conclusions

In conclusion, the results of the present study align with the existing evidence that UC patients, especially those with active disease, present with significant lung functional abnormalities. This is accompanied by increased inflammatory markers in the exhaled air, suggesting subclinical lung inflammation associated with IBD activity. The evidence of distal airway involvement, as demonstrated by elevated CANO levels and the reduction in the efficiency of the pulmonary alveolocapillary diffusion mechanism, underscores a complex interplay between autoimmune diseases and the pulmonary interstitium, leading to small-airway damage. This distally localized form of damage is likely caused by circulating factors and immune complexes, ultimately leading to increased bronchial inflammation, which affects the proximal or large airways. Thus, while larger prospective studies are necessary to confirm the presence of this subclinical entity, it seems that IBDs like UC exhibit characteristics of a multisystemic inflammatory disease affecting numerous systems, including the lungs. In clinical practice, only a small proportion of UC patients are identified as suffering from pulmonary dysfunction. Therefore, clinicians should be vigilant in recognizing early signs of lung dysfunction. In this regard, pulmonary function testing through volume and DLCO measurements may constitute an affordable, easily accessible, and non-invasive method for the early detection of latent lung involvement. As newer non-invasive methods advance, including the measurement of FeNO and markers of oxidative stress, and possibly the inclusion of induced sputum for cytological analysis, these techniques may provide us with additional information about inflammatory status in specific patients. This, in turn, can help us better understand the causes behind pulmonary diseases. In UC patients, our findings emphasize the significance of routine assessments using an integrated approach combining lung function assessment with the non-invasive monitoring of airway inflammation through exhaled NO measurements. Overall, we strongly advocate for close collaboration between gastroenterologists and pulmonologists to enhance the effectiveness of therapy through a robust multidisciplinary approach.

## 6. Limitations

A limitation of our study is the relatively small sample size. Furthermore, numerous confounding factors could potentially influence our FeNO analysis. Given that all the patients were already receiving anti-inflammatory therapies and biologics at the time of enrolment, it becomes challenging to precisely differentiate between alterations that may have existed prior to treatment and those possibly related to therapy. These variations may, in fact, correspond to distinct stages in each patient’s clinical and treatment history. Lastly, we did not perform bronchoprovocation tests or collect any radiological data.

## Figures and Tables

**Table 1:** Clinical characteristics of the study population. Dichotomous values are expressed as numbers and percentages, while continuous values are represented as mean values ± std, medians, and interquartile ranges (IQRs). Disease activity is denoted using the Mayo score, with values of 1 indicating mild disease, 2 reflecting intermediate disease, and 3 signifying severe disease.

Variable	All, *n* = 83	UC, *n* = 42	Controls, *n* = 41	*p*-Value
Age, years				
Median (IQR)	32 (28–37)	36 (31–43)	29 (27–32)	<0.0001
Mean ± std	34.3 ± 10.9	39.0 ± 13.3	29.5 ± 3.8	
Male sex,				
*n* (%)	39 (46.9)	22 (52.4)	17 (41.4)	0.32
Disease activity, *n* (%)		42 (100)		
Mild (1)		3 (7.1)		
Intermediate (2)		13 (31)		
Severe (3)		26 (61.9)		

UC—ulcerative colitis.

**Table 2:** Lung function and inflammatory marker (CRP, FeNO, CANO) measurements in controls and UC patients. Dichotomous values are presented as numbers and percentages, while continuous values are displayed as mean values ± std, medians, and interquartile ranges (IQRs).

Variable	All, *n* = 83 Median (IQR) Mean ± Std	UC, *n* = 42 Median (IQR) Mean ± Std	Controls, *n* = 41 Median (IQR) Mean ± Std	*t*-Test *p*-Value	Multivariate Linear Regression Model	
					Beta Estimated (CI 95%)	*p*-Value
Lung function						
FVC, %	94 (90–98) 93.9 ± 7.4	92 (88–96) 91.3 ± 6.7	96 (92–99) 96.5 ± 7.3	0.0009	−3.3 (−6.60; −0.00038)	0.0500
FEV1/FVC, %	84 (81–87) 83.3 ± 4.9	84 (78–88) 82.0 ± 5.9	84 (82–87) 84.5 ± 3.1	0.019	−0.67 (−2.81; 1.47)	0.5300
FEV1, %	97 (93–103) 98.0 ± 9.6	95 (92–99) 95.8 ± 9.6	97 (94–104) 100.2 ± 9.2	0.04	−1.42 (−5.79; 2.96)	0.5200
TLC, %	96 (86–104) 95.4 ± 12.1	90 (82–98) 92.0 ± 11.1	98 (90–106) 98.8 ± 12.2	0.01	−1.45 (−6.55; 3.64)	0.5700
DLCO, %	90 (78–103) 91.9 ± 16.8	78 (73–82) 79.7 ± 10.6	102 (96–112) 104.4 ± 12.1	<0.0001	−21.46 (−26.79; −16.14)	<0.0001
Inflammatory markers						
CRP, mg/dL	NA	12.9 (6.1–35.5) 24.1 ± 23.3	NA			
FeNO, ppb	10.8 (7.9–18.4) 15.4 ± 11.8	18.3 (6.7–34.1) 20.4 ± 14.7	10.1 (8.2–12) 10.3 ± 3.4	<0.0001	9.17 (3.92; 14.41)	0.0008
CANO, ppb	2.4 (1.8–6.1) 4.3 ± 3.9	5.9 (2.1–10) 6.3 ± 4.5	2.1(1.5–2.5) 2.2 ± 1.0	<0.0001	4.01 (2.40; 5.62)	<0.0001
					*	

UC—ulcerative colitis; FVC—forced vital capacity; FEV1—forced expiratory volume in one second; TLC—total lung volume; DLCO—diffusing capacity for carbon monoxide; %—percentage of predicted; CRP—C-reactive protein; FeNO—fractional exhaled nitric oxide; CANO—alveolar concentration of nitric oxide; *—Adjusted by sex, age.

**Table 3:** Lung function, CRP, and exhaled nitric oxide measurements in the UC patient subgroup by disease activity according to Mayo score. Data are presented as mean values ± std, medians, and interquartile ranges (IQRs).

Variable	Mild (1–2), *n* = 16 Median (IQR) Mean ± Std	Severe (3), *n* = 26 Median (IQR) Mean ± Std	*t*-Test	Multivariate Linear Regression Model	
			*p*-Value	Beta Estimated (CI 95%)	*p*-Value
Age, years	35.0 (32.5–38.0) 39.6 ± 16.3	37.0 (30.0–43.0) 38.7 ± 11.5	0.8400		
BMI, ratio	21.5 (19.3–25.9) 22.1 ± 3.6	21.5 (19.3–25.9) 22.9 ± 2.2	0.3900		
FeNO, ppb	6.2 (4.5–8.8) 7.7 ± 5.7	27.1 (18.4–38.4) 28.1 ± 12.9	<0.0001	20.43 (13.40; 27.47)	<0.0001
CANO, ppb	2.1 (1.4–2.9) 2.7 ± 2.3	8.2 (6.1–11.0) 8.6 ± 4.1	<0.0001	5.80 (3.49; 8.11)	<0.0001
CRP, mg/dL	5.9 (2.0–16.1) 10.4 ± 11.4	23.0 (12.0–45.0) 32.5 ± 24.9	0.0020	22.68 (9.24; 36.12)	0.0020
TLC, %	98.0 (89.5–102.0) 95.9 ± 12.01	88.0 (80.0–98.0) 89.6 ± 9.9	0.0700	−6.81 (−11.99; −1.63)	0.0100
DLCO, %	84.5 (76.0–93.5) 84.8 ± 14.5	77.5 (72.0–80.0) 76.6 ± 5.7	0.0100	−8.71 (−14.28; −3.15)	0.0030
				*	

UC—ulcerative colitis; TLC—total lung volume; DLCO—diffusing capacity for carbon monoxide; %—percentage of predicted; CRP—C-reactive protein; FeNO—fractional exhaled nitric oxide; CANO—alveolar concentration of nitric oxide. *—Adjusted by sex, age, Mayo 3 vs. 1–2.

## Data Availability

The data presented in this study are available from the authors. For any further requests, contact the corresponding author.

## Abbreviations

IBD—inflammatory bowel disease; UC—ulcerative colitis; CAI—colitis activity index; FeNO—fractional exhaled nitric oxide; CANO—alveolar concentration of nitric oxide; CRP—C-reactive protein; PFTs—pulmonary function tests; FEV1—forced expiratory volume in one second; FVC—forced vital capacity; TLC—total lung volume; DLCO—diffusing capacity for carbon monoxide; %—percentage of predicted; ATS—American Thoracic Society; ERS—European Respiratory Society; COPD—chronic obstructive pulmonary disease; ILD—interstitial lung disease.
